# Gold nanoclusters: Photophysical properties and photocatalytic applications

**DOI:** 10.3389/fchem.2022.958626

**Published:** 2022-07-19

**Authors:** Dajiao Cheng, Rong Liu, Ke Hu

**Affiliations:** Department of Chemistry, Fudan University, Shanghai, China

**Keywords:** gold nanoclusters, photophysics, photocatalysis, solar energy conversion, phototherapy

## Abstract

Atomically precise gold nanoclusters (Au NCs) have high specific surface area and abundant unsaturated active sites. Traditionally, Au NCs are employed as thermocatalysts for multielectron transfer redox catalysis. Meanwhile, Au NCs also exhibit discrete energy levels, tunable photophysical and electrochemical properties, including visible to near infrared absorption, microsecond long-lived excited-state lifetime, and redox chemistry. In recent years, Au NCs are increasingly employed as visible to near infrared photocatalysts for their high photocatalytic activity and unique selectivity. This review focuses on the photophysical properties of a variety of Au NCs and their employment as photocatalysts in photocatalytic reactions and related applications including solar energy conversion and photodynamic therapies.

## Introduction

**FIGURE 1:**
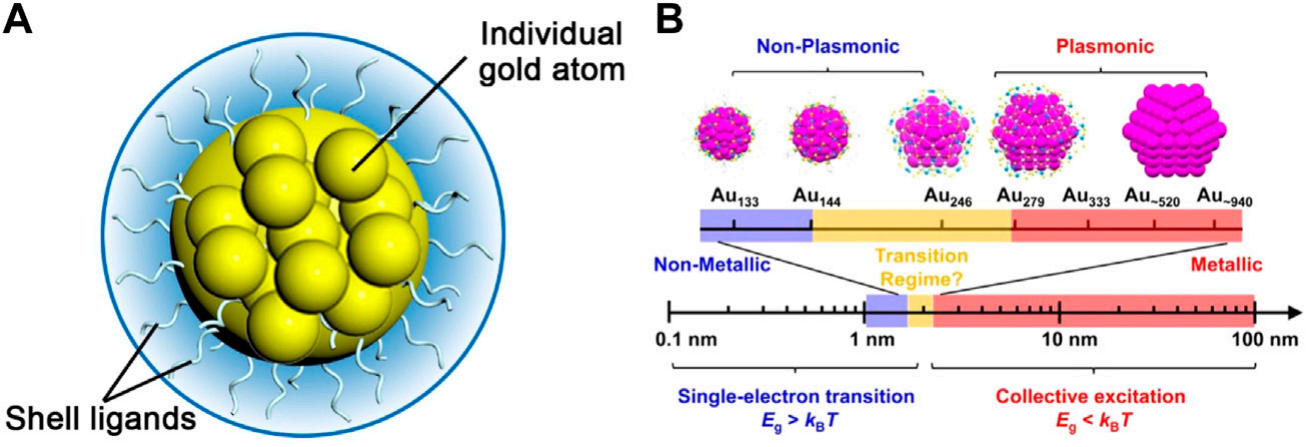
**(A)** Schematic illustration of the core-shell structure of Au NCs **(B)** Changes in the band gap as the number of atoms in a material decreases. Reprinted with permission from (Zhou et al., 2021). Copyright 2021 American Chemical Society.

Metal nanoclusters (NCs) are a new type of nanomaterials with core-shell structures. The core comprises a few to hundreds of metal atoms and the outer shell consists of ligands protecting the core from aggregation (Figure 1A) (Chen and Li, 2020). In recent decades, noble metal nanoclusters especially gold have attracted extensive attention. The size of gold nanoclusters (Au NCs) is typically less than 2 nm, which is between that of small molecules and metal nanoparticles (NPs). Because the size is close to the de Broglie wavelength of the electron at the Fermi level of Au atoms (about 0.5 nm) (Xu and Suslick, 2010), Au NCs exhibit discrete energy levels and a variety of molecular-like properties (Aikens, 2011), such as the atomically precise molecular formula, multiple visible absorption peaks, tunable luminescence, and molecular-like excited-state dynamics (Figure 1B) (Song et al., 2016; Maity et al., 2019; Zhou et al., 2021).

Unlike small molecules, Au NCs possess high specific surface areas and a large number of unsaturated reaction sites (Du et al., 2020), which lead to high catalytic activities and unique selectivity in catalytic reactions such as oxidation, hydrogenation, and C-C coupling (Li and Jin, 2013; Fang et al., 2016; Jin et al., 2016). It is worth mentioning that monodisperse Au NCs with well-defined structures can be used as model catalysts to reveal the correlation between catalyst performance and structure at the atomic scale (Du et al., 2020). The focus of this review will be on the optical properties from Au NC’s unique electronic transitions as well as the practical applications of photocatalysis, solar energy conversion, and phototherapies that make use of Au NC’s electronically excited states.

## Photophysical properties

### Basic theories of gold nanocluster structure

The photophysical properties of atomically precise gold nanoclusters are closely related to the number of gold atoms. Researchers found that Au NCs had a specific number of atoms, known as the “magic number” (Negishi et al., 2004; Negishi et al., 2007). The magic-number gold nanoclusters exhibit high stability and similar photophysical properties. The “superatom electronic theory” proposed by Häkkinen et al. explained the stability of magic-number gold nanoclusters (Hakkinen, 2008; Walter et al., 2008). The core of Au NCs has delocalized ‘‘superatomic orbitals’’ including 1S, 1P, 1D, 2S, 1F, 2P, 1G, 2D, 1H, 3S, 1I.... The effective valence electrons of the gold core are filled into superatomic orbitals in turn according to the aufbau rule. Closed electron shells appear for the gold cores similar to the chemically inert noble gases, so Au NCs are chemically stable. For example, Au_102_(SR)_44_ protected by organic thiolate ligands (-SR), the first Au NCs to be comprehensively characterized by X-ray crystal structure determination, has the exact formula of Au_79_ [Au (*p*-MBA)_2_]_19_ [Au (*p*-MBA)_3_]_2_ (Jadzinsky et al., 2007). The effective valence electron number of the Au_79_ core is 58 (= 79-19-2), which just meets the closed-electron-shell configuration of 1S^2^1P^6^1D^10^2S^2^1F^14^2P^6^1G^18^, so the Au_79_ core exhibits high stability (Walter et al., 2008).

The correlation between the photophysical properties and the number of gold atoms is attributed to the discrete energy levels, which are affected by the quantum size effect. The quantum size effect was explained in detail by the Kubo criterion and the Jellium model (Kubo, 1962; Wood and Ashcroft, 1982).

Kubo proposed that the gap between the highest occupied state and the lowest unoccupied state (called the Kubo gap δ) of a particle consisting of N atoms was proportional to *E*_f_/*N*, where *E*_f_ is the Fermi potential of the bulk metal. Comparing *d* and thermal energy (*k*T), it is possible to judge whether a material has metallic or non-metallic properties (Kubo, 1962). When *d* is less than *k*T, electrons are excited thermally to generate free electron-hole pairs. However, when *d* is much larger than *k*T, free electrons are confined to the discrete energy levels (Roduner, 2006). Metal nanoclusters are usually non-metallic with molecular-like photophysical properties.

Later, the Jellium model clarified the quantitative relationship between the electronic structure of the cores in the metal nanoclusters and the number of metal atoms. In the Jellium model, the valence electrons of the metal core are confined in orbitals that have the same symmetry as the atomic orbitals. The valence electrons are also filled according to the aufbau rule. D. M. Wood and N. W. Ashcroft proved that the energy gap of metal nanoclusters was about *E*_f_/*N* ^1/3^ through the Jellium model, where *N* was the number of metal atoms in the cluster and *E*_f_ was the Fermi potential energy of the bulk metal (Wood and Ashcroft, 1982).

### UV-vis absorption

Ultrasmall-sized gold nanoclusters with discrete energy levels exhibit unique optical absorption, radiative transitions, and excited-state dynamics (Aikens, 2011). Researchers delved into the origin and influencing factors of these photophysical properties, including the cluster geometry such as size, crystal structure and atomic packing, the charge state of the cluster, and the nature of the ligands.

**FIGURE 2:**
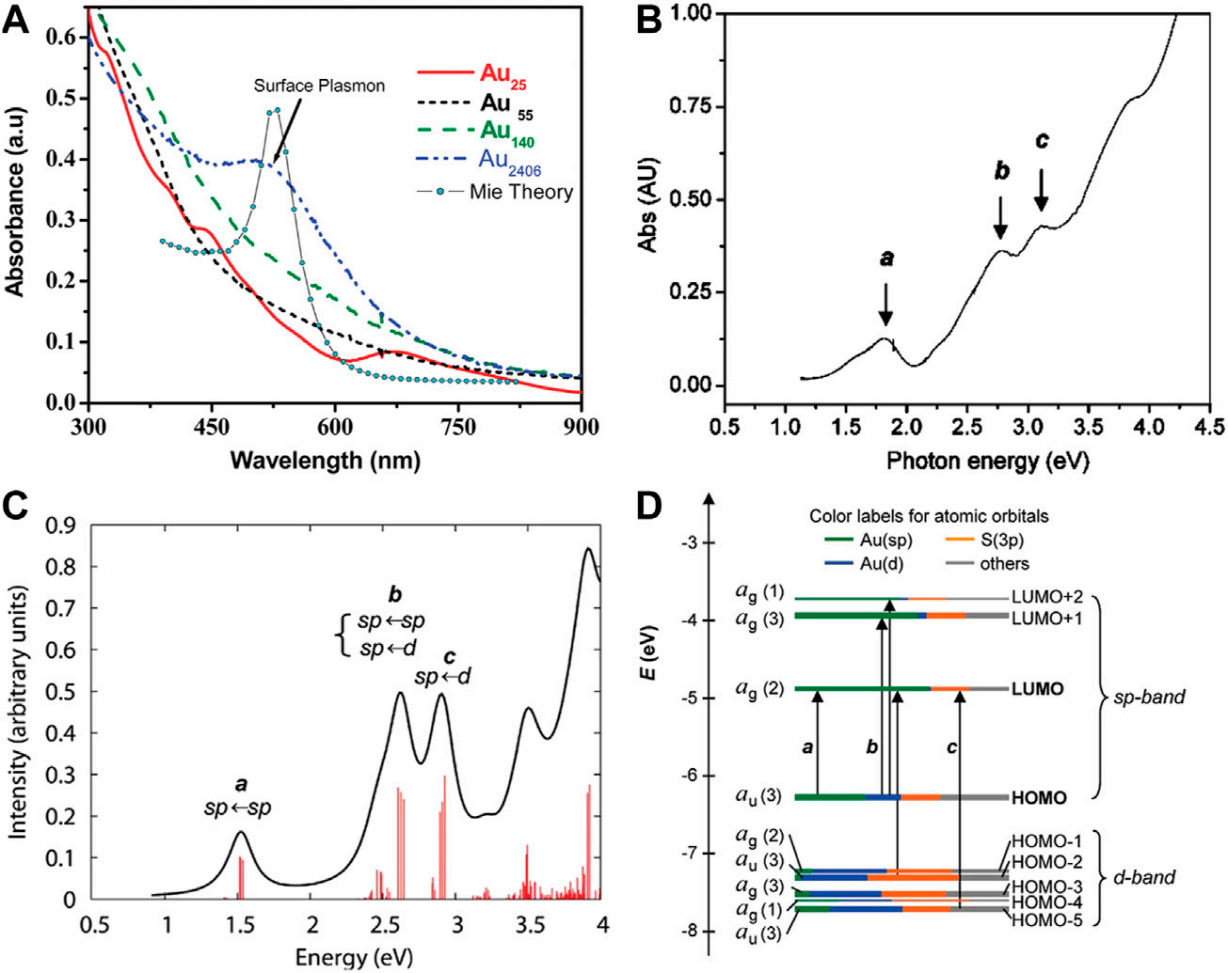
**(A)** UV-vis-NIR absorption spectra of gold nanoparticles and gold nanoclusters. Reprinted with permission from (Yau et al., 2010). Copyright 2010 American Chemical Society **(B)** The experimental absorption spectrum and **(C)** the theoretical absorption spectrum of Au_25_(SH)_18_^-^ **(D)** Kohn–Sham orbital energy level diagram for a model compound Au_25_(SH)_18_^−^. Reprinted with permission from (Zhu et al., 2008a). Copyright 2008 American Chemical Society.

The optical absorption spectra of gold nanoparticles and gold nanoclusters have obvious differences. Gold nanoparticles such as Au_2406_ NPs have the surface plasmonic resonance peak, while Au NCs do not have (Figure 2A) (Yau et al., 2010). The single-electron transitions between discrete energy levels of Au NCs constitute the molecular absorption (Aikens, 2011).

In 2008, Jin and co-workers first reported the crystal structure of Au_25_(SR)_18_ and studied the correlation between the electronic structure and optical properties by time-dependent density functional theory (TDDFT) calculations (Zhu et al., 2008a). The theoretical absorption spectrum of Au_25_(SH)_18_^-^ is in good agreement with the experimental absorption spectrum (Figures 2B,C). Peak a is attributed to the HOMO-LUMO intraband transition (sp←sp). Peak c corresponds to the interband transition (sp←d) from HOMO-n to LUMO. Peak b belongs to the mixed transition (Figure 2D).

Crystal structure analysis shows that the center of Au_25_(SR)_18_ is an icosahedral Au_13_ core and the outer layer is composed of twelve Au atoms and eighteen thiolate ligands. Each outer Au-Au pair (six pairs in total) is bridged by one-SR ligand and with two other-SR ligands bridging between the outer Au atoms and the icosahedral Au_13_ core. HOMO, LUMO, LUMO+1, LUMO+2 and other orbitals are almost entirely composed of the atomic orbitals of thirteen Au atoms in the icosahedral core. Therefore, the absorption peak a at 1.52 eV (Figures 2B,C) is regarded as a transition caused by the electronic structure and geometry of the Au_13_ core (Zhu et al., 2008a). Biicosahedral [Au_25_(PPh_3_)_10_(SC_*n*_H_*2n+1*_)_5_Cl_2_]^2+^ clusters have the completely different crystal structure (Shichibu et al., 2007). The optical absorption peak at 1.76 eV originates from the interaction between two icosahedral Au_13_ units sharing vertices rather than the electronic transitions within a single Au_13_ unit. The above two findings indicate that the crystal structure of Au NCs also affects the optical absorption.

Although the Au_25_(SR)_18_ cluster is divided into two parts including the Au_13_ core and the ligand layer, the optical absorption spectrum of Au_25_(SH)_18_^-^ cluster is not a simple linear combination of the spectra from the metal core and the ligand, which is demonstrated with more detailed TDDFT calculations. The complex absorption spectrum of the Au_25_(SR)_18_ cluster arises from the geometrical and electronic interactions between the metal core and ligands (Aikens, 2008).

**FIGURE 3:**
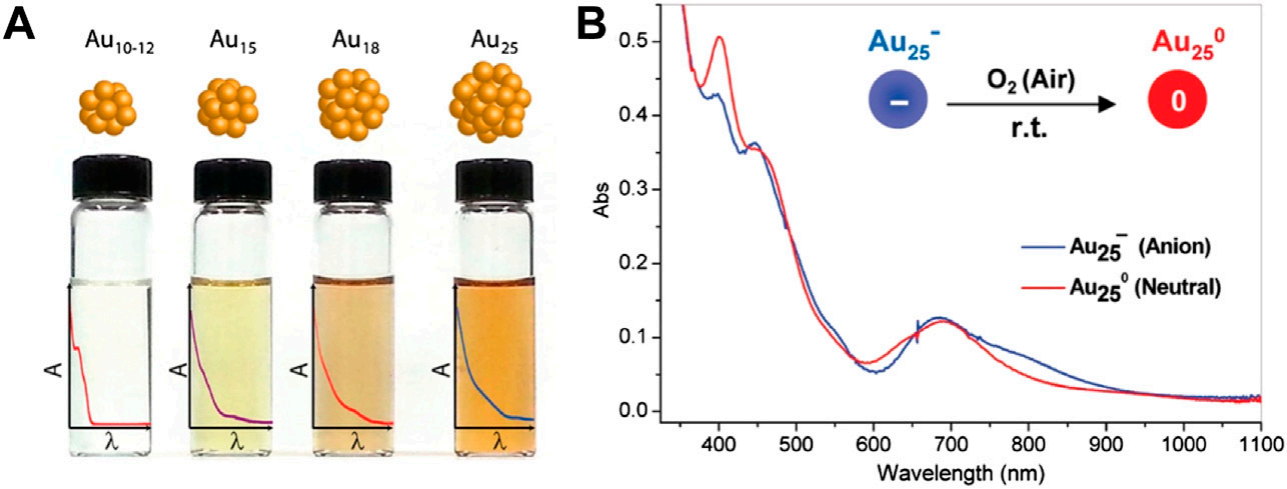
**(A)** The structural diagram and the optical absorption spectra changes of different sizes of Au NCs. Reprinted with permission from (Stamplecoskie and Kamat, 2014). Copyright 2014 American Chemical Society **(B)** Optical absorption spectra of anionic Au_25_^−^ cluster (blue) and charge neutral Au_25_ cluster (red) in solution. Reprinted with permission from (Zhu et al., 2008b). Copyright 2008 American Chemical Society.

Optical absorption of Au NCs is affected by the quantum size effect. As the size of the Au NCs decreases, the gap of discrete energy levels increases and the optical absorption of the clusters shows blue shifts (Figure 3A). For example, the absorption onsets of Au_10-12_(SG)_10-12_ cluster, Au_15_(SG)_13_ cluster, Au_18_(SG)_14_ cluster, and Au_25_(SG)_18_ cluster are 450, 650, 700, and 900 nm, respectively (Yu Y. et al., 2013; Stamplecoskie and Kamat, 2014). The absorption onset of the Au_144_(SR)_60_ cluster is located at 700 nm (Qian and Jin, 2011). Meanwhile, when the cluster size is reduced to Au_25_(SR)_18_, the absorption onset shows the blue shift to 670 nm. Jin summarizes the stable sizes of thiolate-protected gold nanoclusters and the size dependence of the optical absorption in detail (Jin, 2015).

However, there are exceptions to the size dependence of the optical absorption. For example, the size of the Au_22_(SR)_18_ cluster is larger than that of the Au_18_(SR)_14_ cluster, but its absorption onset shows a blue shift of 20 nm (Yu et al., 2014; Pyo et al., 2015; Yu et al., 2015). This phenomenon suggests that the optical absorption of Au NCs is not only related to the size but also to the ratio between the number of ligands and the number of metal atoms.

The electron-withdrawing properties of surface-protecting ligands in Au NCs also affect the optical absorption of the Au NCs. For example, the introduction of electron-withdrawing groups (-X) into p-thiophenol (p-SPhX) ligands induces distortion to the Au_25_(SPhX)_18_ framework and the degree of distortion increases with enhancement of the electron-withdrawing properties. As the distorted degree of the cluster framework increases, the HOMO-LUMO gap becomes smaller and the optical absorption exhibits red shifts (Tlahuice-Flores et al., 2013). A similar phenomenon is also observed in the Au_38_(SPhX)_24_ cluster, where the optical absorption exhibits a slight red shift with the enhancement of the electron-withdrawing properties of the -X substituent. However, Murray and co-workers believe that the ligand layer affects the electron energy of the gold core in the cluster but does not change the HOMO-LUMO energy gap (Guo and Murray, 2005). The HOMO-LUMO electronic absorption peak of the Au_25_(SNap)_18_^-^ cluster (SNap = 1-naphthalenethiolate) shows a red shift of about 10 nm compared with that of the Au_25_(SC_2_H_4_Ph)_18_^-^ cluster, which is attributed to the expansion of the Au_13_ core induced by the aromatic thiolate ligands (Li et al., 2016).

Au NC surface charges from surface-protecting ligands also affect the optical absorption of Au NCs. Xie et al. used multiple ligands with different charges, such as −COO^-^, −NH_3_^+^, and −OH, to induce the surface charge anisotropy of the Au_25_ clusters. This results in structural distortion, which in turn leads to the anomalous optical absorptions at about 780 and 980 nm (Yuan et al., 2016).

In addition to the charges of the ligands, the surface charge states of the clusters also have an effect on the optical absorption spectra (Lee et al., 2004; Negishi et al., 2007; Zhu et al., 2008b). For example, anionic Au_25_^−^ cluster loses one electron and counterion tetraoctylammonium TOA^+^ when it is oxidized to charge neutral Au_25_ cluster in air, resulting in the structural distortion of the gold core which affects the optical absorption of the cluster. The optical absorption spectra of the anionic cluster and the charge neutral cluster are generally similar in shape. However, the absorption around 800 nm in the anionic cluster disappears in the charge neutral cluster, while the weak absorption around 400 nm in the anionic cluster is significantly enhanced in the charge neutral cluster (Figure 3B) (Zhu et al., 2008b). When the Au_25_(SC_2_H_4_Ph)_18_^q^ cluster is oxidized, the surface charge *q* gradually changes from negative charge to positive charge, resulting in an enlarged HOMO-LUMO gap and a red shift in the optical absorption (Venzo et al., 2011). Density functional theory (DFT) calculations further verify the correlation between the surface charge states of the cluster and the optical absorption (Antonello et al., 2013).

### Photoluminescence

**FIGURE 4:**
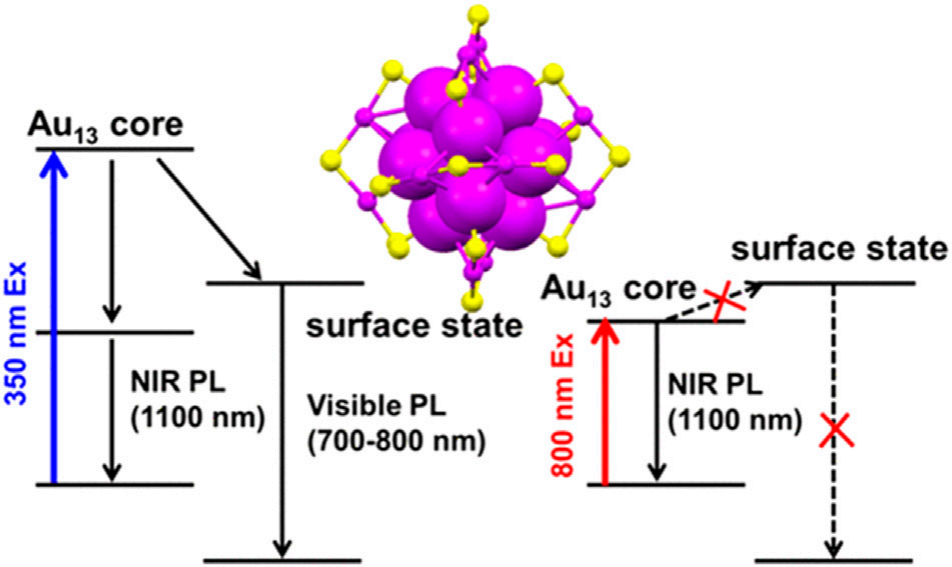
Schematic illustration of the photoluminescent origin of Au_25_ clusters. Reprinted with permission from (Zhou and Song, 2021). Copyright 2021 American Chemical Society.

Au NCs exhibit visible to near-infrared (NIR) emission (Bigioni et al., 2000; Huang and Murray, 2001; Link et al., 2002). The origin of Au NCs photoluminescence remains debatable. Ramakrishna et al. employing time-resolved luminescence spectroscopy of monolayer thiolate-protected Au_25_ clusters have revealed that the NIR luminescence originates from the S-Au-S-Au-S semi-ring states and the visible luminescence originates from the Au_13_ core states (Devadas et al., 2010). However, Aikens et al. calculated that photoluminescence of the Au_25_ cluster were all ascribed to core-based orbitals. Variation of the ligand lengths only influenced photoluminescence spectra from molecular orbital energy shifts. (Weerawardene and Aikens, 2016). A more recent study of the Au_25_ clusters photoluminescence asserted that NIR emission around 1,100 nm should be the dominant gap emission of the Au_13_ core state rather than the weak emission around 700 nm of the core-shell charge transfer state because the photoluminescent excitation spectra with emission monitored at ∼700 nm showed a different profile in comparison with the absorption spectra of Au_13_ clusters. In addition, the lifetime of the Au_25_ clusters excited state obtained from transient absorption spectroscopy is highly consistent with the lifetime of that obtained from the NIR photoluminescence. (Figure 4) (Zhou and Song, 2021). The detailed photoluminescence origin of the Au NCs is subject to data interpretation possibly due to different charge-donating capability or hydrophilicity of ligands. The changes under different experimental conditions may also vary significantly.

In earlier studies, the quantum yields (QY) of most thiolate-protected gold nanoclusters are less than 1% and the utilization of incident light is low (Link et al., 2002; Negishi et al., 2004; Negishi et al., 2005). A few Au NCs exhibit higher quantum yields including the Au_18_(SG)_14_ cluster with a QY of 5.3% and the Au_22_(SG)_18_ cluster with a QY of about 8% (Ghosh et al., 2012; Yu et al., 2014). Researchers find that the size and structure of Au NCs determine the photoluminescence emission wavelength and QY. Meanwhile, both different surface-protecting ligands and aggregation-induced emission (AIE) can enhance QY.

**FIGURE 5:**
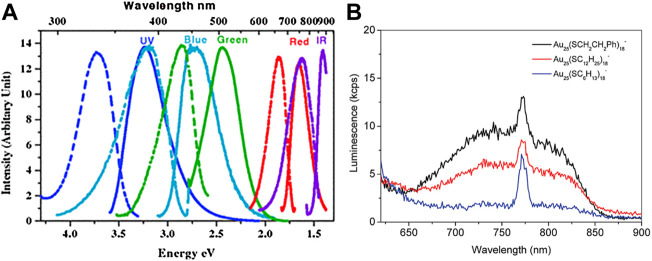
**(A)** Excitation spectra (dashed) and emission spectra (solid) of dendrimer-protected gold nanoclusters different sizes. Reprinted with permission from (Zheng et al., 2004). Copyright 2004 by the American Physical Society **(B)** Photoluminescence spectra of Au_25_(SR)_18_^−^ with different −R groups (−C_2_H_4_Ph, −C_12_H_25_, −C_6_H_13_). Reprinted with permission from (Wu and Jin, 2010). Copyright 2010 American Chemical Society.

Bulk metals with no band gap and non-radiative decay usually have weak photoluminescent emission. As the size shrinks to the nanometer scale and approaches the Fermi wavelength of electrons, the bulk metal transforms into the cluster. The discrete energy levels of clusters cause strong luminescence in addition to the molecular-like optical absorption. According to the Jellium model (Wood and Ashcroft, 1982), as the atom numbers in the metal core of the cluster increase, there is a red shift in the photoluminescence. For example, pepsin-protected Au_8_ cluster, Au_13_ cluster, and Au_25_ cluster synthesized by changing the pH emit blue, green, and red photoluminescent emission, respectively (Kawasaki et al., 2011). Adjusting the molar ratios among the dendrimer ligands, Au^3+^, and the amount of NaBH_4_, a series of Au_5_ clusters, Au_8_ clusters, Au_13_ clusters, Au_23_ clusters, and Au_31_ clusters of the same type were synthesized and their photoluminescent emission bands shift from UV to NIR (Figure 5A) (Zheng et al., 2004).

Optical properties of Au NCs are not only related to the size but also to the crystal structure. Biicosahedral [Au_25_(PPh_3_)_10_(SC_*n*_H_*2n+1*_)_5_Cl_2_]^2+^ clusters and the icosahedral Au_25_(SC_2_H_4_Ph)_18_ have completely different crystal structures (Zhu et al., 2008a). Although both clusters have 25 gold atoms, they have different UV-vis absorption and photoluminescent emission.

The photoluminescence of Au NCs is also affected by the surface ligands, which have little effect on the photoluminescence wavelength but significantly affect QY. Ligands with electron-rich atoms (such as N, O) or groups (such as −COOH, NH_2_) can interact with the surface of Au NCs and transfer delocalized electrons directly to the Au core to effectively enhance QY. For example, water-soluble thiolate GSH-protected Au_25_(SG)_18_^−^ clusters show stronger photoluminescence emission than hydrophobic organic thiolate-protected Au_25_(SR)_18_^−^ clusters (Wu and Jin, 2010).

Electron donating ligands can enhance ligand-to-metal charge transfer (LMCT) and ligand-to-metal-metal charge transfer (LMMCT) to strengthen the photoluminescent emission intensity. Jin and co-workers find that the photoluminescence intensity of hydrophobic thiolate-protected gold nanoclusters is proportional to the electron-donating properties of the ligands (Figure 5B) (Wu and Jin, 2010). In the follow-up work, Jin et al. verify the conclusion again that the lower electronegativity or the stronger electron-donating property of the ligands have, the stronger the photoluminescence of the Au_36_(SR)_24_ clusters are (Kim et al., 2017). The decreased electronegativity of the cluster can also strengthen the electron transfer between the ligand and the gold core to enhance photoluminescent emission. The higher valence of the surface charge *q* the Au_25_(SC_2_H_4_Ph)_18_^q^ cluster has, the stronger photoluminescence intensity the cluster shows (Wu and Jin, 2010).

Polar ligands can also affect the electron transfer between thiolate ligands and the gold core to change the photoluminescence intensity of the cluster. For example, an increase in the polarization of the Au-S bond can enhance the photoluminescence intensity. Murray et al. find that when the non-polar ligands on the surface of Au_38_ clusters and Au_140_ clusters are replaced by polar ligands, the NIR photoluminescence intensity of the clusters increases and the intensity is related to the number of polar ligands linearly (Wang et al., 2006).

**FIGURE 6:**
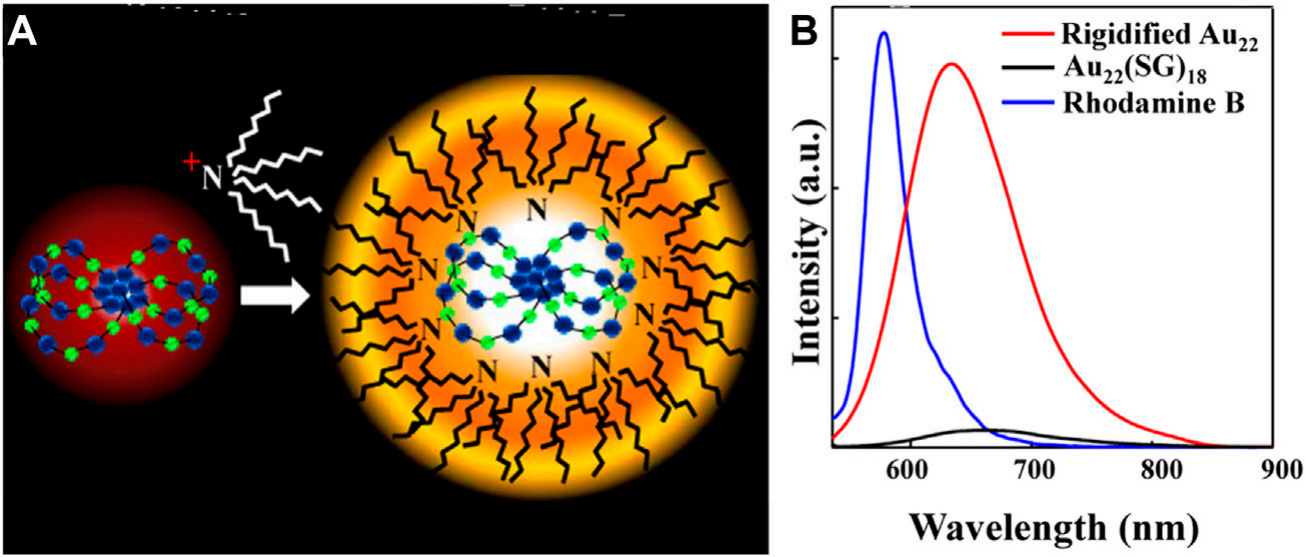
**(A)** Schematic representation of TOA^+^ binding to the Au_22_(SG)_18_ cluster (Au in blue and S in green) **(B)** Photoluminescence spectra of Au_22_(SG)_18_ clusters in aqueous solution and TOA−Au_22_ clusters in toluene. Reprinted with permission from (Pyo et al., 2015). Copyright 2015 American Chemical Society.

The photoluminescence intensity of non-photoluminescent polydentate polymer-protected gold nanoclusters is proportional to the electron-donating ability of the ligands and is also affected by the steric hindrance of the polymer (Li et al., 2013). Au NCs protected by sterically hindered polymer PTMP-P*t*BMA have the highest QY of about 20.1%. The rigid Au(I)-thiolate shell can also improve the QY of the clusters (Pyo et al., 2015). The QY of the Au_22_(SG)_18_ cluster is increased to 60% after the gold shell was rigidified by TOA^+^ (Figure 6).

Aggregation-induced emission can significantly enhance the luminescence intensity of Au NCs. Xie and co-workers have synthesized ultrabright Au (0)@Au(I)-thiolate core-shell nanoclusters (Luo et al., 2012). The addition of a weakly polar solvent disrupts the hydration shell and neutralizes the surface charge so that the short Au(I)-thiolate motifs aggregates on the Au (0) core. Conventional Au(I)-thiolate NCs, which originally do not emit light in aqueous solution, emit strong photoluminescence after aggregation with a QY of about 15%.

### The excited state dynamics

The excited state dynamics of Au NCs are so unique that they differ significantly from gold nanoparticles. Firstly, the excited-state lifetime of Au NCs (∼ns) is much longer than that of gold nanoparticles (∼70 fs) (Varnavski et al., 2001; Zhou et al., 2017b). Typically, Jin et al. reported the excited state lifetime of body-centered cubic Au_38_ cluster is about 5 microseconds (Zhou et al., 2019). Furthermore, high pump power prolongs the electron-phonon coupling time of gold nanoparticles. (Zhou et al., 2016). However, the excited state dynamics of Au NCs is independent on the pump laser intensity, which is similar to the properties of molecules (Yau et al., 2010; Stamplecoskie and Kamat, 2014; Zhou et al., 2016). Au_55_ NCs exhibit a molecular-like single-electron relaxation process. The transient absorption at 640 nm of the Au_55_ clusters decays from the initial state to the intermediate state quickly and then decay back to the ground state slowly (Varnavski et al., 2010; Yau et al., 2010).

At present, it is generally accepted that the core-shell relaxation model is used to analyze the excited state dynamics of Au NCs. When the Au_25_(SR)_18_^−^ cluster is excited, the ultrafast charge relaxation at the Au_13_ core is less than 200 fs and the relaxation from the gold core state to the semi-ring state is about 1 ps (Miller et al., 2009; Devadas et al., 2010). Goodson and Kamat et al. find that the excited state dynamics of Au NCs are mainly affected by the properties of the S-Au-S-Au-S semi-ring state through transient absorption spectroscopy (Devadas et al., 2010; Yau et al., 2010; Stamplecoskie et al., 2014).

**FIGURE 7:**
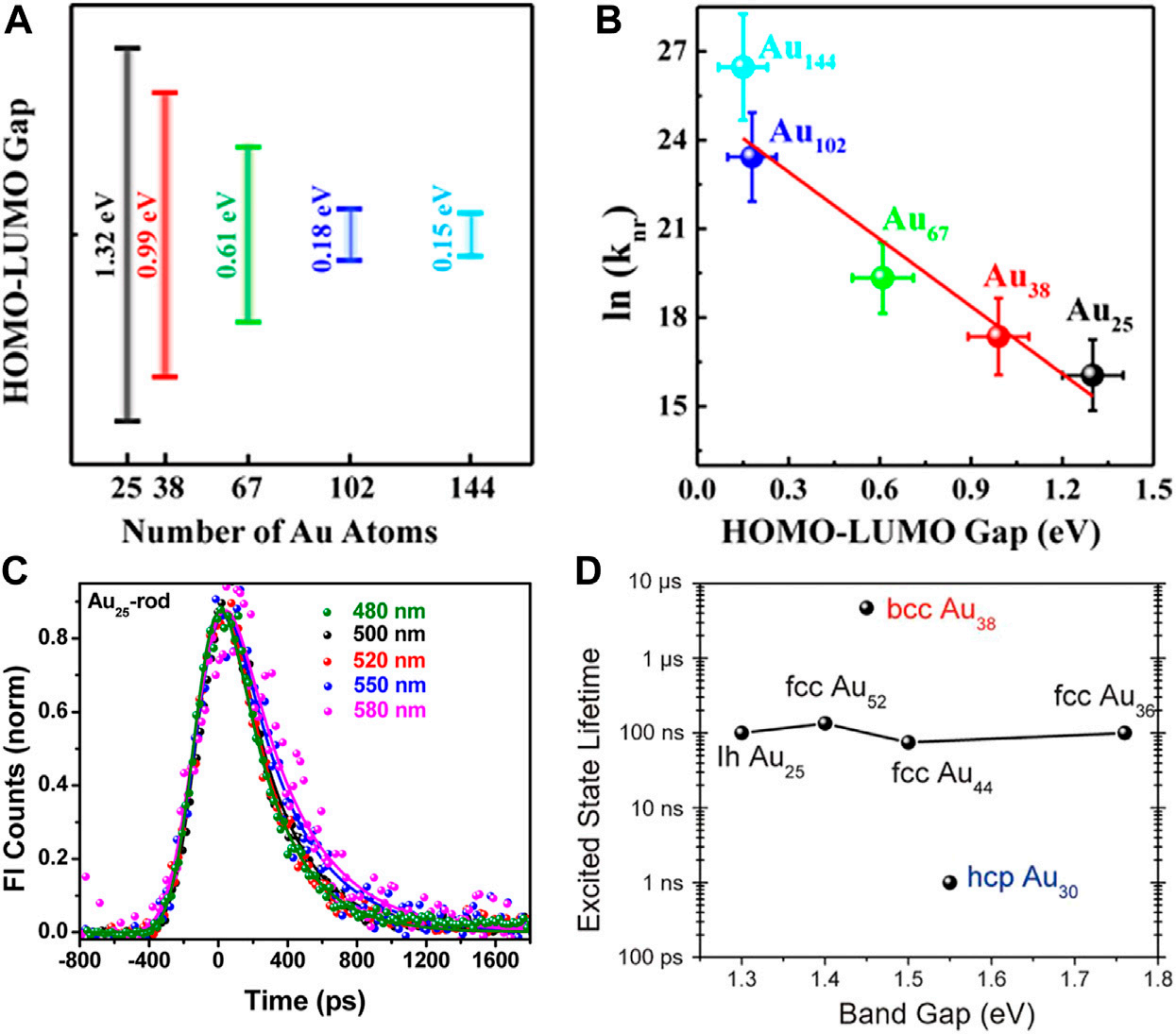
**(A)** Plot of the HOMO-LUMO energy gap versus the number of Au atoms for Au NCs **(B)** Plot of the logarithmic form of the nonradiative decay transition rate (referred to as ln (k_nr_)) versus HOMO-LUMO gap. Reprinted with permission from (Kwak et al., 2017). Copyright 2017 American Chemical Society **(C)** Luminescence kinetic decay traces of Au_25_-rod at different excitation wavelengths. Reprinted with permission from (Devadas et al., 2013). Copyright 2013 American Chemical Society **(D)** Plot of the Excited state lifetime versus band gap of bcc Au_38_, hcp Au_38_, and fcc Au_36_/Au_44_/Au_52_. Reprinted with permission from (Zhou et al., 2019). Copyright 2019 American Association for the Advancement of Science.

As mentioned previously, the photophysical properties of Au NCs are highly dependent on the size. Lee and Ramakrishna et al. observe that the excited state lifetimes of Au NCs also show the distinct size dependence. As the size of Au NCs decreases or the number of Au atoms decreases, the enlarged HOMO-LUMO gap leads to the reduced nonradiative decay transition rate and the increased exciton lifetime (Figures 7A,B) (Kwak et al., 2017). This phenomenon indicates that the excited state dynamics of Au NCs can be explained by the energy gap law, which states that the nonradiative decay dynamics are inversely proportional to the energy gap (Englman and Jortner, 1970). However, Kamat et al. find that Au_18_(SR)_14_ cluster has the longest excited state lifetime among Au_10-12_(SR)_10-12_ cluster, Au_15_(SR)_13_ cluster, Au_18_(SR)_14_ cluster, and Au_25_(SR)_18_ cluster (Stamplecoskie and Kamat, 2014). This result deviates from the expectation of the energy gap law. The explanation is that Au_18_(SG)_14_ cluster and Au_25_(SG)_18_ cluster with the gold core exhibit fast relaxation of less than 1 ps and long-lived LMCT of about 200 ns, while small-sized Au NCs only show long-lived LMCT without fast relaxation processes in the gold core.

The structure of how the gold atoms arrange inside the cluster also affects its excited state dynamics. Ramakrishna et al. compared the femtosecond luminescence dynamics of Au_25_-rod clusters, Au_25_-sphere clusters, and Au_38_-rod clusters. They find that the luminescence kinetic decay traces of Au_25_-rod are independent of the excitation wavelength (Figure 7C), while the kinetic growth traces and the kinetic decay traces of Au_25_-spheres and Au_38_-rods show the specific excitation wavelength dependence, which is attributed to the cascade relaxation from core-gold to shell-gold (Devadas et al., 2013). Jin et al. studied two structural isomers of Au_38_(SC_2_H_4_Ph)_24_ in which the Au_23_ core of the Au_38Q_ cluster was the bi-icosahedron and that of the Au_38T_ cluster wass made up of a mono-icosahedral Au_13_ capped by a Au_12_ tri-tetrahedron by sharing two atoms (Zhou et al., 2017a). The Au_38Q_ cluster shows a rapid decay of 1.5 ps followed by nanosecond relaxation back to the ground state. The excited state dynamics of the Au_38T_ cluster is similar to that of the Au_38Q_ cluster except that the rapid decay process is accelerated by about 50%. The picosecond decay originates from the core-shell charge transfer or the electron rearrangement in the gold core, so it is speculated that the accelerated picosecond decay in the Au_38T_ cluster is attributed to its unique core structure.

Jin et al. also find that the atomic packing of Au NCs also greatly affects its excited state dynamics (Zhou et al., 2019). As shown in Figure 7D, the HOMO-LUMO gaps of face-centered cubic (fcc) Au NCs with different sizes vary greatly, but the excited state lifetimes hardly change. The excited state lifetime of hexagonal close-packed (hcp) Au_30_(SR)_18_ cluster is reduced by about two orders of magnitude and that of body-centered cubic (bcc) Au_38_S_2_(SR)_20_ cluster is increased by nearly two orders of magnitude. This significant excited state lifetime difference is related to the distance and their connection modes between the Au_4_ tetrahedral units.

The surface charge state of the cluster also affects its excited state dynamics in addition to the geometry and size. Taking the ultrafast electron relaxation dynamics of Au_25_(SR)_18_^*q*^ (*q* = 0, −1) as an example, the core excitation lifetime of the anionic cluster is about 1,000 times longer than that of the neutral cluster (Qian et al., 2010). The excited state relaxation of Au_25_(SC_2_H_4_Ph)_18_^*q*^ (*q* = 0, −1) mainly originates from the nonradiative energy transfer from the gold core to the ligands. The energy is transferred from the LUMO orbital to the ligands in the neutral cluster, while the energy is transferred from the LUMO+1 orbital above the LUMO orbital for the anionic cluster and the excited state lifetime is extended by about a hundred times (Green and Knappenberger, 2012).

**FIGURE 8:**
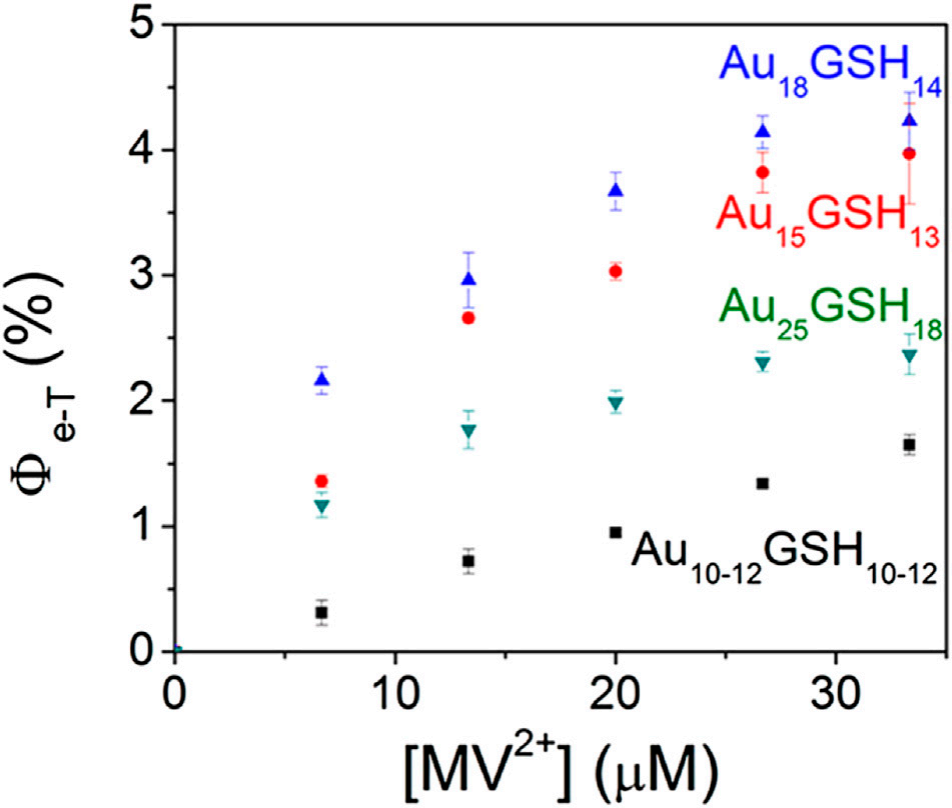
Quantum efficiency of electron transfer, Φ_e-T_, following the 355 nm laser pulse excitation of aqueous Au_x_GSH_y_ solution containing different concentrations of (MV^2+^). Reprinted with permission from (Stamplecoskie and Kamat, 2014). Copyright 2014 American Association for the Advancement of Science.

Besides the excited state lifetime, the electron transfer ability of the excited state is also crucial for the material to become a potential photosensitizer. Methyl viologen [(MV^2+^)] is often used as an electron acceptor to examine the photoinduced electron transfer kinetics in Au NCs and its correlation with photocatalytic activity. Kamat et al. find that only long-lived semi-ring states (or LMCT states) of Au NCs are involved in electron transfer processes (Stamplecoskie et al., 2014). Therefore, the effect of different ligands on the LMCT states can tune the electron transfer efficiency of Au NCs. Meanwhile, Marcus theory suggests that the electron transfer rate decreases rapidly as the distance between the donor and acceptor becomes longer (Silverstein, 2012). Theoretically, the larger the shell ligands are, the smaller the electron transfer rates of Au NCs become (Chen and Li, 2020). Kamat et al. also demonstrate that the electron transfer efficiency is highly correlated with the size of Au NCs. As the size of clusters decreases, the electron transfer efficiency increases (Figure 8) (Stamplecoskie and Kamat, 2014). Au NCs with high electron transfer efficiencies and good visible light absorption have the potential to be excellent photosensitizers (Abbas et al., 2016).

## Photocatalytic applications of gold nanoclusters

The current society is facing the dual pressure of economic development and environmental protection. Photocatalysis is an environmentally friendly technology that can realize solar energy conversion and has broad development prospects. Suitable photocatalysts enable efficient and selective photocatalytic reactions and their optical properties have a great influence on the catalytic efficiency and selectivity (Du et al., 2020). Good photocatalysts have the following characteristics: 1) broad visible light absorption to maximize sunlight utilization and gain selectivity; 2) long-lived photoinduced charge separated states to react with substrate molecules before relaxation to the ground states; 3) high reduction or oxidation potentials to react with a range of substrates; 4) the ability to transfer photogenerated electrons and holes to the reactive interface efficiently (Chai et al., 2019; Witzel et al., 2021).

As mentioned above, Au NCs with the ultra-small size and atomically precise composition exhibit high specific surface area, abundant unsaturated active sites, discrete energy levels, and controllable electronic properties. Therefore, Au NCs have received extensive attention in the fields of thermocatalysis and electrocatalysis (Fang et al., 2016; Chai et al., 2019; Du et al., 2020). Au NCs serving as promising photocatalysts have also raised increasing interest. With the in-depth study of the synthetic methods and optical properties of Au NCs, the energy level gap and the band position of Au NCs can be tuned by adjusting the size, crystal structure, atomic packing, surface charge state or surface ligands. The change of the energy level gap and the band position affect the optical absorption, the separation lifetime of photogenerated electron-hole pairs and the redox ability. At present, Au NCs have been used in photocatalytic conversion (oxidation and reduction) of the organics, photocatalytic degradation of the organic dyes, photocatalytic water splitting, photocatalytic CO_2_ reduction and phototherapy.

### Photocatalytic conversion of organics to value added products

Reactive oxygen species (ROS) are a class of chemically active molecules containing oxygen including singlet oxygen (^1^O_2_), superoxide, hydrogen peroxide (H_2_O_2_), and hydroxyl radicals (•OH). Photosensitizers can generate ROS through the photochemical reactions, which are mainly divided into two reaction pathways (Foote, 1991). In the type I reaction pathway, various oxygen radicals, such as superoxide and hydroxyl radicals, are generated by the electron transfer from the excited state photosensitizers to the substrates. In the type II reaction pathway, the photosensitizer is excited to form the singlet excited state (S_1_) and then transformed into the triplet excited state (T_1_) through the intersystem crossing. The triplet photosensitizer generates ^1^O_2_ by the energy transfer with triplet oxygen (^3^O_2_).

The photosensitizers are usually organic dye molecules with strong absorption or nanomaterials such as quantum dots (QDs) (Samia et al., 2003; Ma et al., 2008), silicon nanocrystals (Kovalev and Fujii, 2005), and metal nanoparticles (Vankayala et al., 2011; Long et al., 2013; Vankayala et al., 2013). Au NCs have been demonstrated to act as photosensitizers and generate ^1^O_2_ efficiently by tuning the electronic structure of the clusters (Kawasaki et al., 2014; Zhang et al., 2018; Du et al., 2020). Au NCs can undergo thermal catalytic oxidation reaction using the organic oxidant PhIO (Li et al., 2012; Liu et al., 2015) or photocatalytic oxidation reaction using ^1^O_2_ generated from energy transfer, the latter being more environmentally friendly.

**FIGURE 9:**
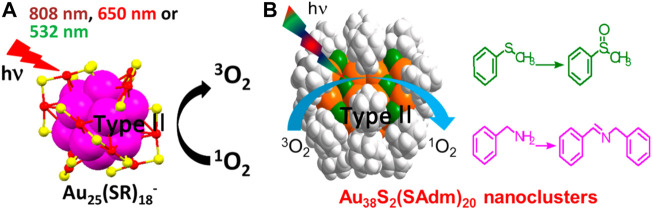
**(A)** Schematic diagram and the characterizations of ^1^O_2_ generation from Au_25_ clusters under the visible or NIR light irradiation. Reprinted with permission from (Kawasaki et al., 2014). Copyright 2014 American Chemical Society **(B)** Au_38_ clusters generate ^1^O_2_ under light and catalyze the oxidation of sulfides to sulfoxides and that of benzylamines to imines selectively. Reprinted with permission from (Li et al., 2017). Copyright 2017 American Chemical Society.

Jin et al. demonstrate that the ability of ^1^O_2_ generation is related to the size of Au NCs (Kawasaki et al., 2014). Regardless of the surface charge state and the solubility, Au_25_ clusters can generate ^1^O_2_ under the visible or NIR light irradiation (Figure 9A). Because of the large HOMO-LUMO gap (1.3 eV), Au_25_ clusters have a high T_1_ yield. In contrast, the HOMO-LUMO gap of Au_38_ clusters is small (0.9 eV) and the energy of T_1_ is lower than that required to excite ^3^O_2_ to form ^1^O_2_ (0.97 eV). Therefore, Au_38_ clusters cannot generate ^1^O_2_. ^1^O_2_ produced by photoexciting Au_25_ clusters can catalyze the oxidation of organic sulfide to sulfoxide with selectivity close to 100%. Besides, Zhu et al. also find that the arylthiolated Au_25_(F-Ph)_18_^−^ nanocluster with NIR emission can photosensitize ^3^O_2_ to produce ^1^O_2_ for photocatalytic oxidative functionalization of sulfide, β-ketoesters, and 2-aryl-1,2,3,4-tetrahydroisoquinoline (Wang et al., 2022).

Maran et al. further investigate the effect of the surface charge state of Au_25_(SC3)_18_^q^ clusters on the photocatalytic performance of ^1^O_2_ generation by time-resolved electron paramagnetic resonance (TR-EPR) (Agrachev et al., 2019). The anion clusters generate ^1^O_2_ under photoexcitation through the type II photoreaction pathway and then the EPR signal is observed. Under the same experimental conditions, neutral clusters cannot generate ^1^O_2_ and no EPR signal is observed.

The above study by Jin et al. shows that Au_38_(SC_2_H_4_Ph)_24_ cannot photo-generate ^1^O_2_ due to the small HOMO-LUMO gap (0.9 eV) (Kawasaki et al., 2014). Li et al. have shown that the energy gap of clusters can be adjusted by changing the atomic packing to improve the photocatalytic performance (Li et al., 2017). The Au_38_S_2_(SAdm)_20_ clusters (−SAdm = 1-adamantanethiolate) are body-centered cubic atomic packing, which is different from the face-centered cubic atomic packing of common Au NCs. The Au_38_S_2_(SAdm)_20_ clusters with the HOMO-LUMO gap of 1.57 eV can photo-generate ^1^O_2_ and the photocatalytic efficiency is higher than that of Au_25_(SR)_18_^−^ described above. In this work, ^1^O_2_ can oxidize not only sulfides to sulfoxides but also benzylamines to imines selectively (Figure 9B).

In summary, Au NCs can be used as photosensitizers to photo-generate ^1^O_2_ for the selective oxidation of organics. In addition, Au NCs can also be combined with semiconductor materials to improve the efficiency of photocatalytic organic conversion under visible light excitation by broadening the optical absorption range or separating photogenerated electron-hole pairs effectively.

**FIGURE 10:**
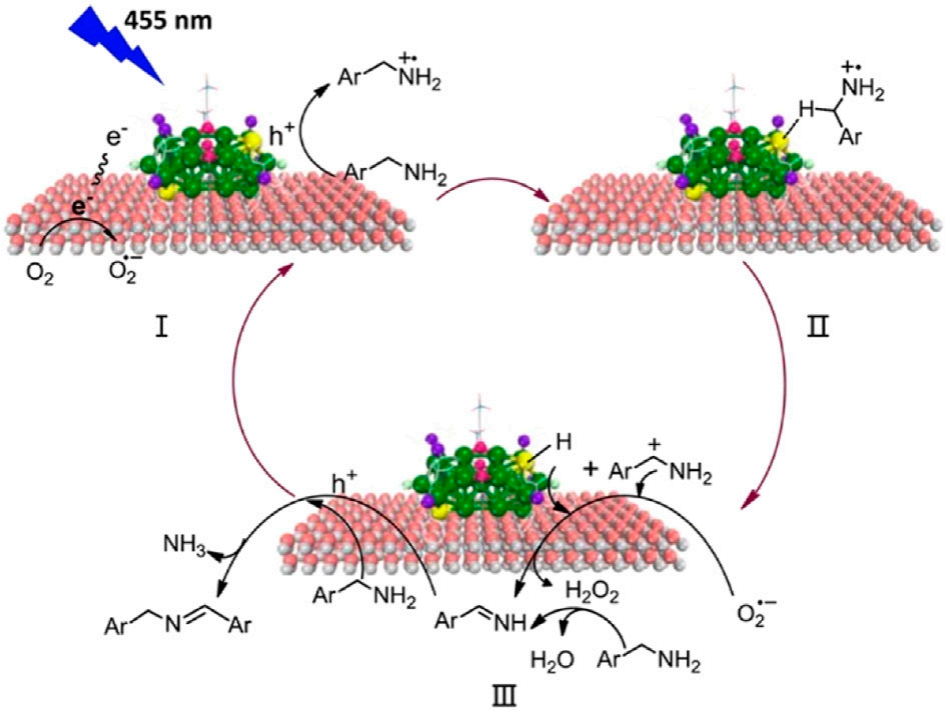
(A) The mechanism diagram of the photocatalytic oxidation of benzylamine by Au_25_/TiO_2_. Reprinted with permission from (Chen et al., 2017). Copyright 2017 American Chemical Society.

Wang and Li et al. synthesized the Au_25_(PPh_3_)_10_(SC_3_H_6_SiO_3_)_5_Cl_2_/TiO_2_ composite for the photocatalytic oxidation of amines to imines efficiently and selectively under mild reaction conditions (Chen et al., 2017). Au_25_(PPh_3_)_10_(SR)_5_Cl_2_ clusters can separate the photogenerated electron-hole pairs effectively leading to the high catalytic activity. The photocatalytic reaction involves an Au-H intermediate and a carbocation intermediate derived from benzylamine (Figure 10), and the reactive sites are the bare Au atoms due to the removal of part of the PPh_3_ ligands.

Liu and co-workers construct Au_x_/NP-TNTA heterostructure by depositing Au_*x*_ clusters onto highly ordered nanoporous layer-covered TiO_2_ nanotube arrays (NP-TNA) *via* a facile electrostatic self-assembly strategy (Xiao et al., 2015). Au_x_/NP-TNTA heterostructures can be used as photocatalysts for the reduction of nitroaromatics. Au_x_ clusters photo-generate electron-hole pairs under sunlight illumination. Because the potential of LUMO in Au_x_ NCs is more negative than the conduction band (CB) edge of TiO_2_ and the interaction between them is tight, the photogenerated electrons of Au_x_ clusters are easily injected into the CB of TiO_2_ to reduce the nitroaromatics adsorbed on the surface of the materials, while the photogenerated holes oxidize ammonium formate as the electron donor. It should be noted that the above reactions occurred in N_2_ to avoid the participation of O_2_. The condition ensured that the nitroaromatics were all reduced by the photogenerated electrons. Au_x_/NP-TNTA heterostructures enhance the performance of photocatalytic reduction of organics through the efficient separation of photogenerated electron-hole pairs.

### Photocatalytic degradation of organic pollutants

**FIGURE 11:**
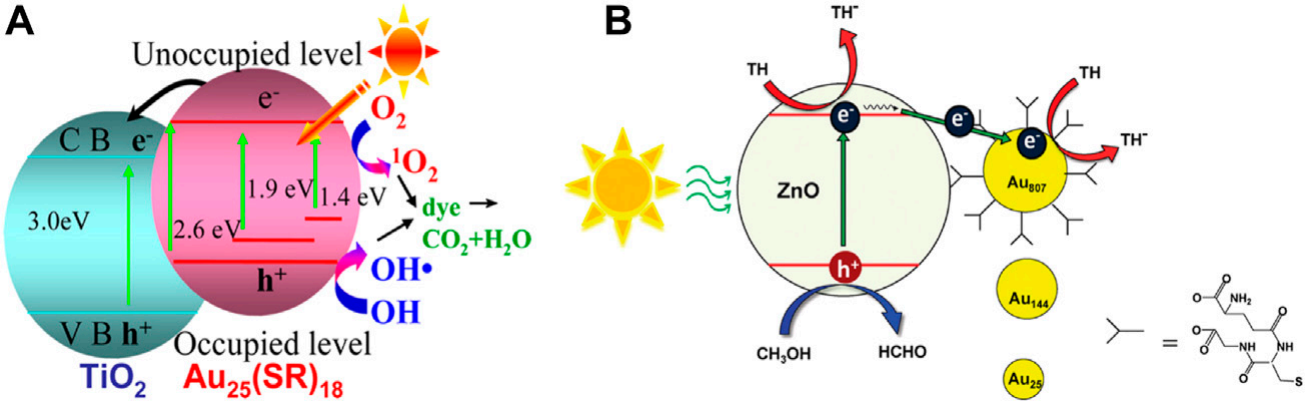
**(A)** Mechanism of the photocatalytic degradation of organic dyes by Au_25_(SC_2_H_4_Ph)_18_/TiO_2_ composites. Reprinted with permission from (Yu C. et al., 2013). Copyright 2013 American Chemical Society **(B)** The photocatalytic degradation of TH of ZnO-Au composites under visible light irradiation. Reprinted with permission from (Lee et al., 2011). Copyright 2011 American Chemical Society.

The composites composed of semiconductors and Au NCs as photosensitizers can also degrade organic dyes through photocatalysis. Jin et al. investigated the performance and mechanism of Au_25_(SC_2_H_4_Ph)_18_/TiO_2_ composites to generate ROS for the degradation of methyl orange (MO) under the visible light irradiation (Figure 11A) (Yu C. et al., 2013). Au_25_(SR)_18_ clusters expand the optical absorption range to NIR region which results in a 1.6-fold increase in the activity of photocatalytic degradation of MO under the visible light irradiation. The photogenerated electrons of Au_25_(SR)_18_ clusters are injected into the conduction band of TiO_2_ to inhibit the recombination of photogenerated electrons and holes effectively, which also contributes to the improvement of the photocatalytic degradation of MO.

Au_x_/NP-TNTA heterostructures constructed by Liu and co-workers are also used for the photocatalytic degradation of MO (Xiao et al., 2015). The photogenerated electrons transferred to the conduction band of TiO_2_ and the photogenerated holes of Au_x_ clusters can react with H_2_O to generate ROS for the degradation of MO. The efficient separation of the photogenerated electron-hole pairs enhances the performance of the photocatalytic degradation of organic dyes.

How to improve the photostability of Au NCs at the interface of composites is a long-standing challenge. Xu and Chen et al. report the SiO_2_-Au GSH clusters-BPEI@TiO_2_ (SABT) composites assembled by a simple method (Weng et al., 2018). Branched polyethyleneimine (BPEI) is not only the surface modifier but also the stabilizer and the reducing agent, which can prevent the oxidation of the ligands on the surface of the clusters. Therefore, the size and structure of Au GSH clusters can be maintained for more than 10 h under the continuous visible light irradiation. The thickness-controllable TiO_2_ coating stabilizes the ultra-small Au GSH clusters supported on the SiO_2_ sphere. As a result, the SABT composite has good photostability and its photocatalytic activity for the degradation of rhodamine B has little change in 10 cycles.

In composites composed of the semiconductor and Au NCs as photocatalysts, Au NCs generally act as photosensitizers to capture the visible light. Au NCs can also accept the photogenerated electrons to catalyze reactions. Both the size and surface ligands of Au NCs affect the efficiency of the photocatalytic degradation of organic dyes.

Three different sizes of Au_x_ (SG)_y_ clusters synthesized by Lee and Song et al. were deposited on the surface of ZnO through the carboxyl groups in their ligands (Lee et al., 2011). It is speculated that the photogenerated electrons of ZnO under visible light irradiation are injected into the Au NCs to reduce thionine (TH) on the surface of the Au NCs. The absorption spectrum of ZnO hardly changes after the cluster deposition indicating that the light-harvesting ability is not enhanced, so the photocatalytic performance is only related to the efficiency of the charge separation. The increased sizes of Au NCs accelerate the electron transfer rate to improve the efficiency of the charge separation (Figure 11B). Therefore, the ZnO-Au composite composed of Au NCs with the large size has higher photocatalytic performance for the degradation of TH.

Xie et al. synthesized thio-β-cyclodextrin (SH-β-CD) protected gold nanoclusters to decorate TiO_2_ (Zhu et al., 2018). TiO_2_-Au NCs@β-CD composites can photocatalyze the degradation of MO efficiently. The mechanism indicates that photogenerated electrons from TiO_2_ under UV light are injected into the gold core to form the catalytic center, which reduces O_2_ to superoxide anion (O_2_^−•^) for the degradation of MO. In addition, the photogenerated holes of TiO_2_ can also oxidize H_2_O to •OH for the degradation of MO. The SH-β-CD ligands on the surface of the clusters can capture MO efficiently through the host-guest interaction. Therefore, the enhanced photocatalytic degradation of MO originates from the synergistic effect of the gold core, SH-β-CD ligands and TiO_2_ in the TiO_2_-Au NCs@β-CD composite.

Wang et al. synthesized the silane-stabilized Au NCs by a simple photoreduction method. The Au NCs act as stand-alone photocatalysts for the photocatalytic degradation of methylene blue (MB) under visible light irradiation (Zhou S. et al., 2017). The silane-stabilized Au NCs degrade about 96% of MB after exposure to the visible light for 1 h. It has been confirmed that the mechanism of the photocatalytic degradation of organic dyes of the silane-stabilized Au NCs is similar to that of semiconductors.

### Photocatalytic water splitting and CO_2_ reduction

**FIGURE 12:**
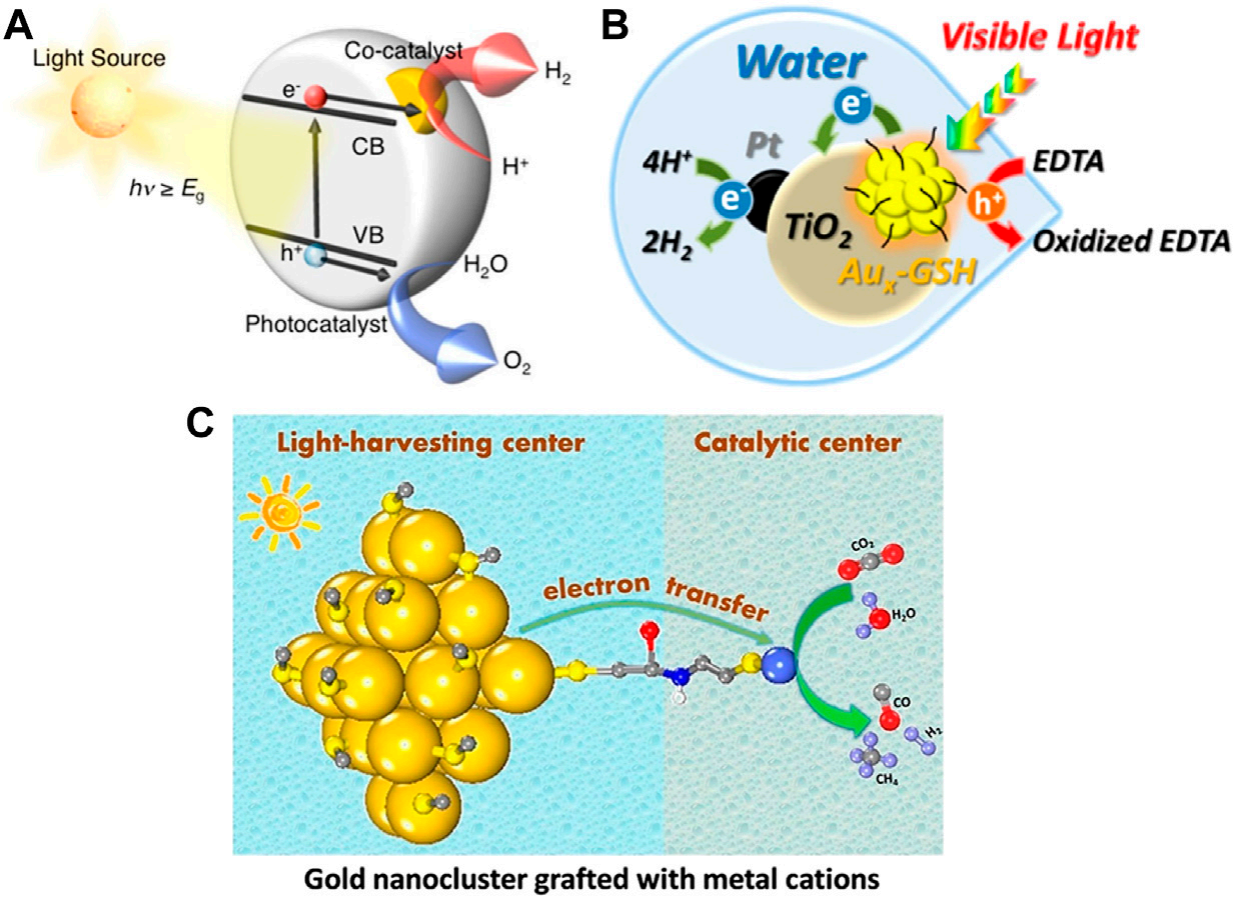
**(A)** Schematic of photocatalytic water splitting by composites *via* a one-step method: conduction band (CB), valence band (VB), band gap (*E*_*g*_). Reprinted with permission from (Negishi et al., 2015). Copyright 2015 American Chemical Society **(B)** Illustration of Au_*x*_-GSH NCs sensitized Pt/TiO_2_ NPs for photocatalytic water splitting reaction. Reprinted with permission from (Chen and Kamat, 2014). Copyright 2014 American Chemical Society **(C)** Schematic of covalently bridged Au-GSH clusters and metal cations for the visible-light-driven CO_2_ reduction. Reprinted with permission from (Cui et al., 2018). Copyright 2018 American Chemical Society.

Au NCs can be used as the cocatalysts in photocatalytic water splitting for the conversion of solar energy to chemical energy. Monodisperse Au_25_(SG)_18_ clusters with unchanged sizes are supported on the BaLa_4_Ti_4_O_15_ photocatalysts and the prepared composites can be used for photocatalytic water splitting (Figure 12A) (Negishi et al., 2013). The experimental results show that the catalytic performance of the Au_25_ cluster cocatalyst is 2.6 times higher than that of the gold nanoparticle cocatalyst. This may be due to the fact that ultra-small Au_25_ clusters can introduce the same number of active sites as the gold nanoparticles at lower loadings to reduce the interference of cocatalysts on the optical absorption of catalysts. It has also been found that the stability of Au NCs precursors is crucial to achieve the controllable loading. Meanwhile, the photocatalytic activity increases as the size of the cocatalyst decreases (Negishi et al., 2015).

Besides acting as the cocatalysts, Au NCs can also be the photosensitizers for H_2_ generation in the photocatalytic water splitting. Kamat and coworkers find that the reversible reduction (E^0^ = −0.63 V vs. RHE) and oxidation (E^0^ = 0.97 and 1.51 V vs. RHE) potentials of glutathione-capped metal nanoclusters (Au_x_-GSH NCs) are suitable for driving water splitting (Chen and Kamat, 2014). Au_x_-GSH NCs sensitized Pt/TiO_2_ NPs in aqueous slurry system can generate H_2_ with sacrificial electron donors, such as EDTA, under the visible light irradiation (Figure 12B). The Au_x_-GSH NCs expand the photoresponse range of the large band gap semiconductor to efficiently realize the conversion of light energy to chemical energy. However, the Au_x_-GSH NCs aggregate into large NPs upon continuous illumination, which limits the photoconversion efficiency.

Au NCs as photosensitizers can also photocatalyze CO_2_ reduction. Xiong and co-workers attach M^2+^ metal cations (Fe^2+^, Co^2+^, Ni^2+^, and Cu^2+^) to Au-GSH NCs covalently through l-cysteine as a bridging ligand to achieve the stable connection between the light-harvesting center and the catalytic site (Cui et al., 2018). The photogenerated electrons are transferred from the Au-GSH NC to M^2+^ *via* the bridge bond and are used to reduce CO_2_ at the catalytic site M^2+^ (Figure 12C). It is noteworthy to overcome the instability of Au-GSH NCs in this reaction system to prevent aggregation.

It has been confirmed that the protection of Au NCs by a metal-organic framework (MOF) can improve the stability of Au NCs in photocatalytic CO_2_ reduction effectively. Recently, Fei et al. prepare N-heterocyclic carbene (NHC)-stabilized ultrasmall Au NCs in a MOF by the heterogeneous nucleation method (Jiang et al., 2021). Au-NC@MOF composites exhibit the stable and excellent catalytic activity for photocatalytic CO_2_ reduction. It has been demonstrated that the photogenerated electrons are transferred from Au NCs to MOFs *via* the MOF-NHC-Au covalent bond bridge to separate photogenerated electron-hole pairs rapidly, which enables the synergistic catalysis of Au NCs and MOFs.

### Phototherapy

Phototherapy is a promising cancer treatment technology with minimal trauma, few side effects and high efficacy (Lin et al., 2021). Photodynamic therapy (PDT) is a clinically licensed non-invasive phototherapy (Dolmans et al., 2003). In the aerobic environment, photosensitizers excited by light with appropriate wavelengths generate ROS through either type I or type II reaction pathways to oxidize adjacent biological macromolecules such as DNA. The photochemical reactions causes the local oxidative damage to kill cancer cells (Li and Grant, 2016).

It has been demonstrated that Au NCs exhibit excellent biocompatibility, pharmacokinetics, renal clearance and biodegradability (Zhang et al., 2012; Zheng et al., 2021). Therefore, Au NCs can be widely used in biomedical fields such as biosensing, photoluminescence imaging, and cancer therapy (Yang et al., 2019; Zheng et al., 2021). At the same time, ultra-small Au NCs exhibit unique optical properties including high two-photon absorption cross section, long-lived triplet state, and high photoluminescence quantum yields. Combining the above two points, Au NCs can be used as photosensitizers in PDT.

Au NCs with high two-photon absorption cross section can improve the efficacy of PDT significantly. Dihydrolipoic acid coated gold nanoclusters (AuNC@DHLA) as photosensitizers can take full advantage of high two-photon absorption and mediate the single electron transfer to generate O_2_^−•^ for the efficient photodynamic therapy (type I) (Han et al., 2020). Meanwhile, two-photon excitation can increase the penetration depth in tissue.

The combination of Au NCs and semiconductors can improve the charge separation efficiency, which also improves the efficacy of PDT significantly. Li and Zhang et al. prepare TiO_2_ NPs-Au NC-graphene composites (TAG) for the efficient PDT (Cheng et al., 2017). Au NCs with narrow band gaps have the cut-off absorption wavelength at 617 nm for the efficient utilization of simulated sunlight. Meanwhile, Au NCs, TiO_2_ NPs, and graphene have staggered energy levels which can separate photogenerated electron-hole pairs effectively. Based on the above two advantages, TAG composites generate a large amount of •OH and O_2_^−•^ *via* the water oxidation and oxygen reduction under simulated sunlight irradiation to induce severe cancer cell death. It demonstrates the significant PDT (type I) efficacy of TAG.

The functional molecules can be bound to the surface of Au NCs covalently through the terminal functional groups, which can also enhance the PDT efficacy of the clusters and expand the applications. A common covalent conjugation scheme is the coupling of carboxyl groups to primary amines *via* 1-ethyl-(3-dimethylaminopropyl)carbodiimide (EDC). For example, a tumor-targeting agent (folic acid) and a photosensitizer (protoporphyrin IX) can be covalently bound to lipoic acid protected Au_18_ clusters to form a multifunctional PFL-AuC nanocomposites, which can be used as a photosensitizer for photoluminescence imaging assisted PDT (type II) with tumor targeting (Nair et al., 2015). PFL-AuC nanocomposite is more efficient for the photo-generation of ^1^O_2_ compared with protoporphyrin IX alone. The tumor-targeting agents cause the local aggregation of PFL-AuC nanocomposites. Therefore, the PFL-AuC nanocomposites can induce PDT to cause the cancer cell death with a low-energy laser. In addition, the NIR emission of the PFL-AuC nanocomposite can track the PDT process in real time. This method of constructing nanocomposites through covalent binding also enables organelle-targeting of Au NCs. For example, the multifunctional nuclear-targeting TAT peptide-Au NCs (peptide sequence: N-GRKKRRQRRR-C) can simultaneously perform photoluminescence imaging, gene delivery, and NIR-excited PDT (type II) to kill cancer cells effectively (Vankayala et al., 2015).

The severely hypoxic environment in solid tumors limits the efficacy of traditional PDT (Li et al., 2018; Liu et al., 2018). Nitroaromatic compounds are bioreductive prodrugs that are activated by nitroreductase (NTR) overexpressed in hypoxic tissues to generate DNA-reactive cytotoxic arylamines, which enables hypoxia-targeting tumor therapy (Searle et al., 2004; Wilson and Hay, 2011). However, the activation of nitroaromatic prodrugs by NTR shows slow reaction kinetics and the regulation of the reaction kinetics is complicated (Searle et al., 2004). In addition, native NTR is difficult to obtain in large quantities and loses its activity easily when pH or temperature changes (Yanto et al., 2010). At present, it has been reported that nanomaterials with catalytic activity can mimic natural enzymes to convert prodrugs into bioactive molecules for cancer treatment (Soldevila-Barreda and Metzler-Nolte, 2019; Oliveira et al., 2020; Vong et al., 2020). Visible light that excites nanomaterials to activate prodrugs is spatially and temporally controllable (Alonso-de Castro et al., 2018; Gurruchaga-Pereda et al., 2019; Zhang and Liu, 2020).

**FIGURE 13:**
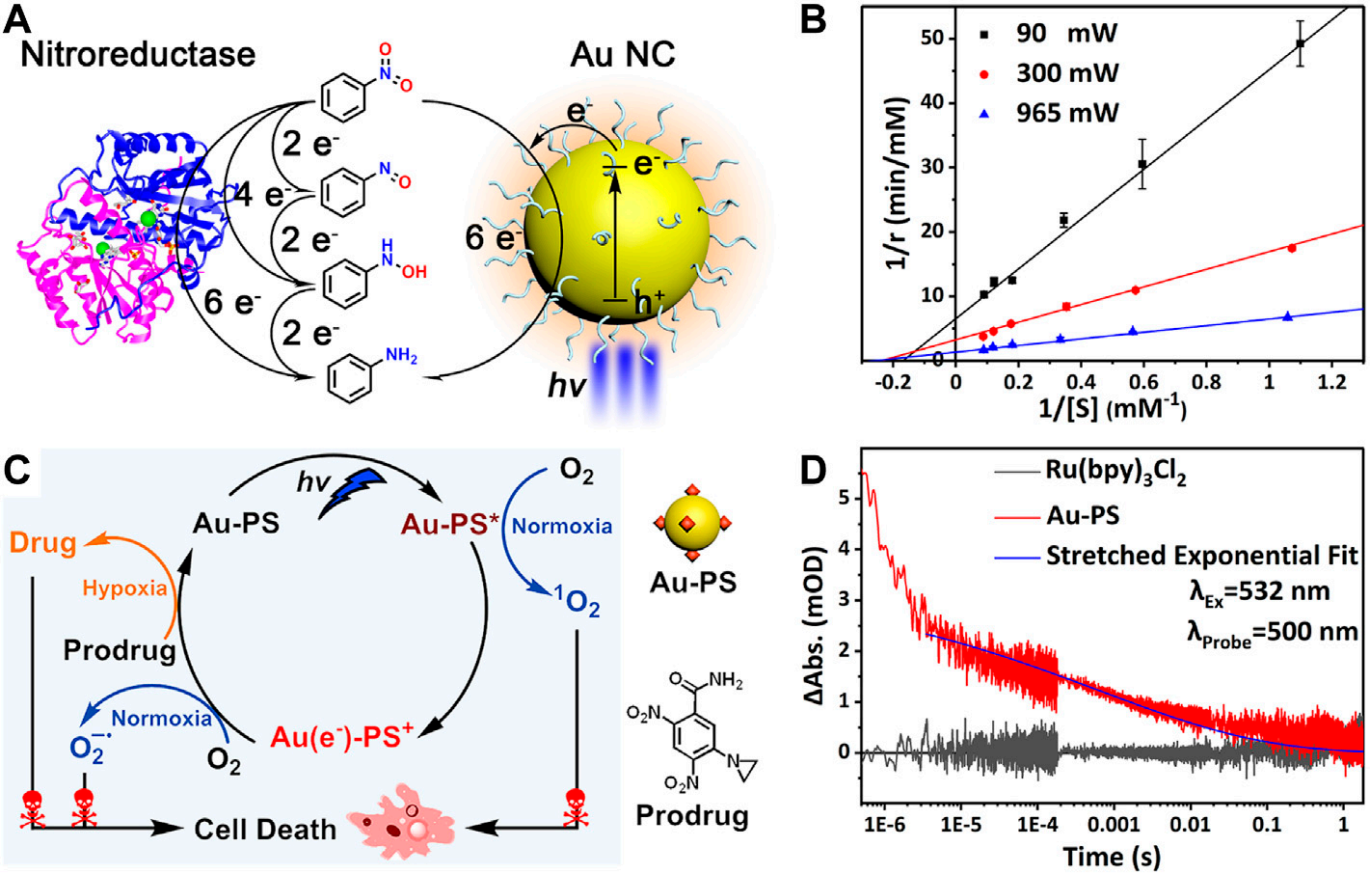
**(A)** Gold nanoclusters perform enzyme-like photocatalysis for nitrobenzene **(B)** Enzyme-like reaction kinetics of gold nanoclusters tuned by the light intensity. Reprinted with permission from (Liu et al., 2021). Copyright 2021 American Chemical Society **(C)** Proposed mechanism of the functionalized gold nanoclusters for photodynamic and photocatalytic double killing of cancer cells **(D)** Time-dependent decay of the transient absorption of charge-separated states in Au-PS. Reprinted with permission from (Cheng et al., 2021). Copyright 2021 American Chemical Society.

Common photosensitizers are difficult to mimic NTR because the reduction of nitroaromatics to arylamines requires a challenging six-electron/six-proton process. Meanwhile, the selective catalytic reduction of nitroaromatics is also a long-standing challenge. Our group used the glutathione protected Au NCs as photosensitizers to mimic NTRs for the selective photocatalytic reduction of nitrobenzene to aniline (AN) in a hypoxic environment (Figure 13A) (Liu et al., 2021). Notably, the catalytic activity of Au NCs can be regulated by the temporally and spatially controllable light (Figure 13B). Finally, Au NCs photocatalyze the activation of nitroaromatic prodrugs in tumor cells to achieve the photochemotherapy against the hypoxic condition of solid tumors, which overcomes the oxygen dependence of the traditional PDT.

However, Au NCs only absorb little blue light and have low utilization of visible light, so the photocatalytic reduction of nitrobenzene is less efficient. In an effort to eliminate this drawback, our group prepared the functionalized gold nanoclusters (Au-PS) as the photocatalyst by covalently linking ruthenium coordination compounds as the photosensitizers to Au NCs (Cheng et al., 2021). In the hypoxic conditions, Au-PS still exhibits the selective photocatalytic reduction of nitrobenzene to aniline under the visible light excitation. At the same time, this functionalization strategy broadens the optical absorption range of the material and prolongs the separation lifetime of photogenerated electron-hole pairs by utilizing the charge transfer from the excited PS to the Au NC core (Figure 13C), so the photocatalytic efficiency is improved significantly. In the normoxic conditions, ROS are generated after the photoexcitation of Au-PS. Therefore, Au-PS can be used both as the photosensitizer for photodynamic therapy in the normoxic conditions as well as being the photocatalyst to activate the nitroaromatic prodrug CB1954 in the hypoxic conditions (Figure 13D). This constitutes a dual kill mechanism to induce the cancer cell death in both normoxic and hypoxic conditions.

## Summary and outlook

The art of synthesizing monodisperse and atomically precise Au NCs makes it possible to finely tune the geometric structure and composition. Au NCs with discrete energy levels exhibit controllable photophysical properties, including optical absorption, redox capacity, and the excited-state lifetime. These tunable photophysical properties give Au NCs great potential for the photocatalytic applications. Although Au NCs can be used for the solar energy conversion, including the degradation of organic pollutants, the conversion of organics, water splitting, and CO_2_ reduction, the photocatalytic reactions of Au NCs are less reported than their applications in thermal catalysis.

In future endeavors of expanding photocatalytic potentials of Au NCs particularly in solar energy conversion, we think a good research direction would be making the best use of the versatile surface chemistry of Au NCs while combining the excellent visible light absorption and charge separation properties. Molecular light harvesters typically do not possess the capability of both light harvesting and multi-electron transfer catalysis whereas Au NCs do. Some organic transformations such as benzylamine oxidation or nitroaromatics reduction discussed in this review have proven Au NCs their potential. With targeted design of the gold core as well as smart choice of Au NC surface-protecting ligands, Au NCs have a bright future as the photocatalysts to utilize solar energy for value added chemical reactions.

In addition to solar energy conversion, Au NCs with good biocompatibility also have great potential as photocatalysts for biomedical application. They can be delivered into mammalian cells for the photocatalytic generation of bioactive molecules. The functionalization of surface ligands can transform Au NCs into targeted multifunctional nanocomposites and enhance the photocatalytic performance in living organisms. So far, reports on Au NC *in vivo* photocatalysis are still very rare. More research is waiting ahead.
